# Advancing fall risk prediction in older adults with cognitive frailty: A machine learning approach using 2-year clinical data

**DOI:** 10.1371/journal.pone.0330672

**Published:** 2025-08-21

**Authors:** Catherine Park, Namhee Kim, Miji Kim, Chang Won Won, Beom-Chan Lee

**Affiliations:** 1 Division of Digital Healthcare, Yonsei University, Wonju, South Korea; 2 Wonju College of Nursing, Yonsei University, Wonju, South Korea; 3 Department of Health Sciences and Technology, College of Medicine, Kyung Hee University, Seoul, South Korea; 4 Elderly Frailty Research Center, Department of Family Medicine, College of Medicine, Kyung Hee University, Seoul, South Korea; 5 Department of Health and Human Performance, University of Houston, Houston, Texas, United States of America; 6 Center for Neuromotor and Biomechanics Research, University of Houston, Houston, Texas, United States of America; National Center for Chronic and Noncommunicable Disease Control and Prevention, Chinese Center for Disease Control and Prevention, CHINA

## Abstract

Falls are a critical concern in older adults with cognitive frailty (CF). However, previous studies have not fully examined whether machine learning models can predict falls in older individuals with CF. The 2-year longitudinal data set from the Korean Frailty and Aging Cohort Study and machine learning approach were utilized to predict fall risk. We analyzed multidimensional health data, including demographics, clinical conditions, as well as the physical and psychological health factors of 443 older adults with CF identified out of 2,404 older adults. For fall risk prediction, we developed a machine learning framework incorporating logistic regression, bootstrapping, and recursive feature elimination. Statistical analysis revealed significant differences between the non-faller and faller groups for nine clinical conditions as well as physical and psychological variables. Using nine significant variables, our machine-learning-based model demonstrated good predictive performance with an area under the curve (AUC) exceeding 80%. Furthermore, our machine learning framework identified four optimal variables: the number of Fried physical frailty (PF) phenotypes, PF-Mobility scores, scores from the Korean version of the Short Geriatric Depression Scale, and scores from SARC-F (consisting of five components: strength, assistance with walking, rising from a chair, climbing stairs, and experiencing falls). It demonstrated excellent predictive performance, with an AUC, sensitivity, specificity, and accuracy exceeding 95%. These variables reflect the critical association between physical and psychological health and fall risk. These findings underscore the importance of integrating multidimensional health data with machine learning methodologies to accurately predict fall risk in older adults with CF, design targeted interventions, and enable healthcare professionals to implement strategies to reduce and prevent such falls.

## Introduction

Falls are a major global health concern in the aging population, particularly among older adults with cognitive frailty (CF) [1]. CF is a clinical syndrome characterized by the simultaneous presence of physical frailty (PF) and cognitive impairment (CI) [2]. Falls can lead to substantial morbidity in older adults with CF, such as fractures, loss of functional independence, and mortality, thereby establishing fall prevention as a critical public health issue [3,4]. Additionally, falls often accelerate aspects of cognitive decline and contribute to adverse health outcomes (e.g., depression and social withdrawal) [5]. The prevalence of falls is higher in older adults with CF than in those without CF. A recent meta-analysis revealed that fall rates in older adults with CF were almost two-fold higher than those in older adults with PF or CI alone, demonstrating the compounded risks associated with this dual condition [6].

Physical and psychological health factors are interdependent and play a crucial role in increasing the risk of falls among older adults with CF. Physical health factors, such as reduced muscle strength, gait abnormalities, and balance impairments, are key contributors to an increased fall risk [7,8]. Similarly, psychological health factors, including depression and anxiety, are strongly associated with an elevated risk [9,10]. Therefore, understanding the complex interplay between these physical and psychological factors has received considerable attention in the design and implementation of effective fall prevention programs for older adults with CF [11,12].

A recent review systematically compared traditional and machine learning-based prediction models for predicting fall risk [13]. Traditional models primarily rely on univariate or basic multivariate statistical analyses that focus on isolated or combinations of risk factors. Although these approaches have provided valuable insights, they often fail to capture the multidimensional complexity of fall risk [13]. Consequently, these models tend to be ineffective in accurately predicting fall risk in diverse populations of older adults [13]. In contrast, machine-learning-based models offer the potential to enhance fall risk prediction by utilizing large data sets and identifying complex and nonlinear data patterns. Compared to conventional statistical approaches, these advanced methodologies process large-scale longitudinal data for more precise and individualized predictions [13]. In addition, machine learning algorithms have outperformed traditional methods in predicting fall risk across diverse older populations, including those with CF [13,14].

A key challenge in improving fall risk prediction models is identifying the optimal variables that contribute most significantly to fall risk. Nevertheless, most previous studies have predominantly focused on physical health factors, with limited or no focus on potential psychological health contributions [14]. A substantial benefit of machine learning techniques is their ability to maintain long-term predictive accuracy through the continual integration of data across multiple domains (e.g., physical and psychological health), with dynamic risk predictions over time [14]. However, existing studies have not fully examined whether machine learning models can predict falls in older individuals with CF over an extended period (≥1 year).

Recognizing these research gaps, we aimed to (1) explore the influence of demographics, clinical conditions, and health status (physical and psychological health) on falls in older adults with CF; (2) develop a machine learning-based model that uses variables extracted from clinical condition information and health status assessments (the baseline assessment) to predict fall risk 2 years after the baseline assessment; and (3) determine the minimum essential variables (optimal variables) required to ensure high accuracy and robustness of the machine learning-based model for fall risk prediction.

## Methods

### Data source and participant selection

We utilized data sets from the first and second waves of the nationwide Korean Frailty and Aging Cohort Study (KFACS) [15]. The data sets were collected during 2016–2017 for the first wave and 2018–2019 for the second wave. The data set, covering the period from 2016 to 2019, included 2,404 community-dwelling older adults aged 70–84 years. Fall-related outcomes were based on self-reported fall incidents collected through in-person interviews conducted in community settings. Participants who were no longer residing in the community at the time of follow-up, such as those who were institutionalized or hospitalized, were typically excluded from the second wave. This reflects the KFACS design, which focuses on community-dwelling older adults.

The study was approved by the Institutional Review Board of Yonsei University (IRB No. 1041849–202411-BM-240–01; approval date: December 9, 2024), and was conducted in accordance with the Declaration of Helsinki. Data were accessed for research purposes on December 10, 2024, following IRB approval. Since this retrospective study used anonymized data, written informed consent was not required under national legislation and institutional guidelines.

**Fig 1:**
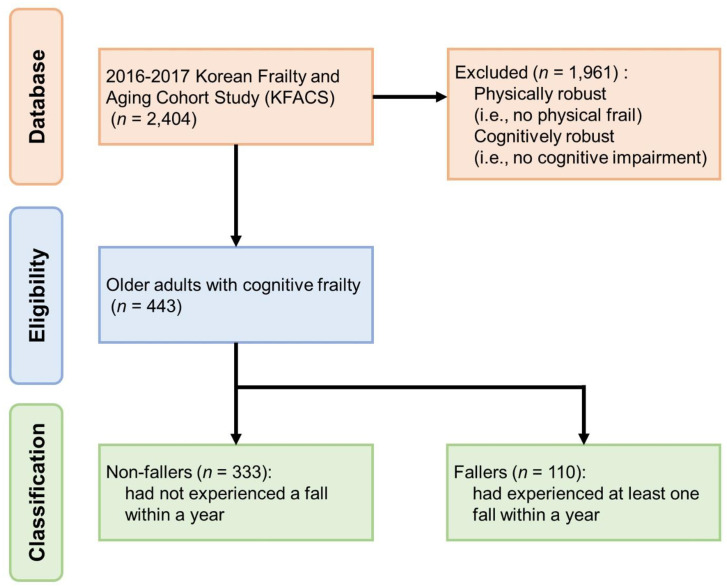
STROBE diagram of eligibility determination and participant group classification.

Among 2,404 older adults, 443 with CF during the first wave were included in this study. The remaining 1,961 participants were excluded because they did not meet the criteria for concurrent PF and CI (Fig 1). CF classification was determined based on the results of the PF and CI assessments available in the data sets. Specifically, participants were classified as having CF if they exhibited one or more of the Fried PF phenotypes [16] and scored <24 on the Mini-Mental State Examination [17], which corresponds to the standard criteria for CF classification [2].

### Group classification and variable extraction

Participants were classified into two groups (non-faller and faller) using the data set for the first wave (i.e., the baseline assessment). The non-faller group included individuals who had not experienced a fall within the year before the baseline assessment. In contrast, the faller group included individuals who had experienced at least one fall during the same period (i.e., within a year before the baseline assessment). The participant selection and group classification processes are illustrated in Fig 1, demonstrating that 333 and 110 participants were classified as non-fallers and fallers, respectively.

Variables associated with participant demographics, clinical conditions, and health status were extracted from the data set of the first wave. The demographic variables included age and sex. Clinical variables included cardiovascular, musculoskeletal, respiratory, cognitive, and polypharmacy. The health status variables were the physical and psychological health factors. Physical health variables were extracted from the results of nine assessments, including (1) Fried PF phenotypes [16]; (2) Mini-Mental State Examination [17]; (3) Korean Activities of Daily Living (K-ADL) scale [18]; (4) PF and Mobility (PF-M) test [19]; (5) five-component Strength, Assistance with walking, Rising from a chair, Climbing stairs, and Falls (SARC-F) questionnaire [20]; (6) Activities-specific Balance Confidence (ABC) scale [21]; (7) sit-to-stand test [22]; (8) Timed Up and Go (TUG) test [23]; (9) the ability to cross a street safely before the traffic light changes was assessed via a self-reported binary item, asking participants whether they were able to cross the street safely within the signal time (Yes/No); and (10) the Mini Nutritional Assessment [24]. Psychological health variables were extracted from the results of three assessments, including the (1) EuroQol Visual Analogue Scale (EQ-VAS) [25], (2) the Korean version of the Short Geriatric Depression Scale (SGDS-K) [26], and (3) fear of falling [27].

### Statistical analysis

Statistical analyses were performed using SPSS Statistics (IBM Corp., Armonk, NY, USA) to evaluate the effects of demographics, clinical conditions, and health status on falls. Categorical variables are presented as numbers and percentages, whereas continuous variables are reported as mean ± standard deviation. Categorical variables were analyzed using the chi-squared test to determine significant differences between the non-faller and faller groups. For continuous variables, the Shapiro–Wilk test was performed to assess their normality. As all continuous variables were not normally distributed, the Mann–Whitney U test was used to compare differences in variables between the non-faller and faller groups. The significance level was set at two-sided *P* < 0.05 for all statistical analyses.

### Machine learning-based model and optimal variable selection

**Fig 2:**
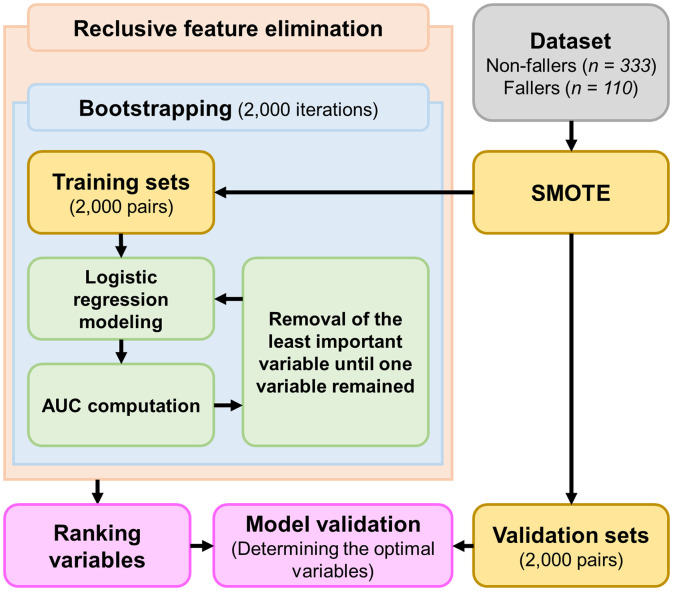
Machine learning framework. AUC, area under the curve; SMOTE, synthetic minority oversampling technique.

The machine-learning framework that we developed is illustrated in Fig 2. The framework was implemented and executed using custom scripts created in MATLAB (MathWorks, Natick, MA, USA). A categorical variable (0 for non-fallers and 1 for fallers) was assigned as the class, whereas another categorical variable (0 for non-falls and 1 for falls, assessed during the second wave for the same participants) was used as the target. The independent variables included significant variables identified through statistical analyses of the demographic, clinical condition, and health status variables. These variables included the presence or absence of osteoporosis, number of Fried PF phenotypes, and scores from the K-ADL, PF-M, SARC-F, ABC, fear of falling, EQ-VAS, and SGDS-K.

Our machine-learning framework uses a logistic regression algorithm combined with bootstrapping and recursive feature elimination, a validated approach for improving model performance [28,29], to develop a machine-learning-based model and determine the minimum essential variables (optimal variables) critical for ensuring accurate fall risk prediction. Considering the number of participants in each group and recommendations from previous studies [30,31], we conducted 2,000 bootstrapping iterations to ensure the robustness of the machine learning-based model. Additionally, to address the class imbalance between the non-faller (n = 333) and faller (n = 110) groups, we applied the synthetic minority over-sampling technique, which randomly generates synthetic samples for the minority class (i.e., the faller group) [32] and created 2,000 training-validation data set pairs for bootstrapping. After each bootstrapping iteration, our machine-learning framework identified and removed the least important variable, as determined by its contribution to the area under the receiver operating characteristic curve, which was repeated recursively until one variable remained (i.e., recursive feature elimination).

We evaluated the performance of the machine-learning-based model using four key metrics: AUC, sensitivity, specificity, and accuracy [28,29,33]. In particular, the AUC quantifies the model’s ability to differentiate between non-falls and falls, while sensitivity and specificity measure the model’s precision in predicting non-falls and falls, respectively. Accuracy represents the overall classification performance of the model. After ranking the variables through recursive feature elimination, we reevaluated the model using the 2,000 validation data set pairs to assess its performance with the reduced variables to determine the optimal variables.

## Results

### Demographics and clinical condition characteristics

Descriptive statistics and statistical analysis results for the demographic data and clinical condition characteristics (i.e., cardiovascular, musculoskeletal, respiratory, cognitive, and polypharmacy) are presented in Table 1. Among all variables, osteoporosis, which was included as an independent variable in the machine learning-based model, showed a significant difference between the groups.

**Table 1:** Demographics and clinical characteristics of the non-faller and faller groups.

Variable	Non-Faller (n = 333)	Faller (n = 110)	*P* value
**Demographics**			
Age, years	77.4 ± 3.8	77.3 ± 3.7	0.698
Sex			
Male, n (%)	108 (32.4)	28 (25.5)	0.209
**Clinical characteristics**			
Cardiovascular condition			
Heart failure, n (%)	2 (0.7)	0 (0.0)	0.586
Peripheral vascular disease, n (%)	3 (1.0)	3 (3.2)	0.174
Cerebrovascular disease, n (%)	14 (4.8)	5 (5.4)	0.200
Musculoskeletal conditions			
Rheumatoid arthritis, n (%)	8 (2.8)	3 (3.2)	0.394
Osteoporosis, n (%)	68 (23.5)	26 (28.0)	<0.0001^*^
Respiratory condition			
Asthma, n (%)	15 (5.2)	5 (5.4)	0.187
COPD, n (%)	2 (0.7)	1 (1.1)	0.728
Bronchitis, n (%)	7 (2.4)	1 (1.1)	0.364
Pulmonary tuberculosis, n (%)	2 (0.7)	0 (0.0)	0.586
Cognitive condition			
Dementia, n (%)	3 (1.0)	1 (1.1)	0.447
Polypharmacy			
Number of prescription medications, n	4.7 ± 3.14	4.4 ± 3.39	0.406

An asterisk (*) indicates statistically significant differences between the groups.

COPD, chronic obstructive pulmonary disease

### Physical and psychological health

Descriptive statistics and statistical analysis results for the physical and psychological health variables are summarized in Table 2. Among all health-related variables (i.e., 13 variables), five physical health variables and three psychological health variables showed significant differences between the groups. The faller group had a significantly higher number of Fried PF phenotypes, higher K-ADL scores, lower PF-M scores, higher SARC-F scores, lower ABC scores, lower EQ-VAS scores, higher SGDS-K scores, and higher fear of falling scores than did the non-faller group (Table 2). These eight significant variables were included as independent variables in the machine-learning-based model.

**Table 2:** Physical and psychological health variables of the non-faller and faller groups.

Variable	Non-Faller (n = 333)	Faller (n = 110)	*P* value
**Physical health**			
Fried PF phenotypes, number (1–5)	1.68 ± 0.8	2.1 ± 1.0	0.001^*^
MMSE, score (0:bad–30:good)	21.4 ± 2.7	21.4 ± 2.6	0.946
K-ADL, score (7:good–21:bad)	7.1 ± 0.4	7.33 ± 0.8	0.015^*^
PF-M, score (0:bad–15:good)	10.7 ± 3.5	9.2 ± 3.6	<0.0001^*^
SARC-F, score (0:good–8:bad)	1.7 ± 1.6	2.3 ± 1.8	0.002^*^
ABC, score (0:bad–100:good)	69.4 ± 21.9	58.8 ± 21.3	<0.0001^*^
Sit-to-stand test, sec	13.2 ± 4.0	13.8 ± 7.6	0.967
TUG test time, sec	11.9 ± 2.8	12.7 ± 3.7	0.152
Cross traffic light turning red, n (%)	305 (91.6)	95 (86.4)	0.156
MNA, score (0:bad–14:good)	12.5 ± 1.7	12.1 ± 2.0	0.076
**Psychological health**			
EQ-VAS, score (0:bad–100:good)	69.3 ± 18.6	64.7 ± 18	0.011^*^
SGDS-K, score (0:good–15:bad)	4.7 ± 4.2	6.5 ± 4.6	<0.0001^*^
Fear of falling, score (1:no–4:fear)	0.38 ± 1.0	1.4 ± 3.1	<0.0001^*^

The asterisk (^*^) indicates statistically significant differences between the groups.

ABC, activities-specific balance confidence; AUC, area under the curve; EQ-VAS, EuroQol visual analogue scale; K-ADL, Korean activities of daily living; MNA, mini nutritional assessment; PF, physical frailty; PF-M, physical frailty and mobility; SARC-F, five-component Strength, Assistance with walking, Rising from a chair, Climbing stairs, and Falls; SGDS-K, Korean version of the short form geriatric depression scale; TUG, timed up and go

### Machine learning-based model performance and optimal variables

The ranking of the nine variables included in the machine learning-based model and validation results using 2,000 validation data set pairs for four performance metrics (AUC, sensitivity, specificity, and accuracy) are presented in Table 3.

**Table 3:** Ranking of nine variables included in the machine learning-based model and validation results of the model using 2,000 validation data set pairs.

Rank	Variable	AUC (95% CI)	Sensitivity (95% CI)	Specificity (95% CI)	Accuracy (95% CI)	Included rank(s)
1	Fried PF phenotypes	85.2% (84.82–85.51)	78.9% (78.39–79.44)	77.7% (77.23–78.17)	78.4% (78.00–78.76)	1
2	PF-M	85.3% (84.99–85.68)	79.4% (78.86–79.92)	78.4% (77.93–78.87)	78.6% (78.24–79.00)	1–2
3	SGDS-K	89.9% (89.55–90.22)	81.3% (80.78–81.73)	90.8% (90.39–91.17)	88.6% (88.24–88.92)	1–3
4	SARC-F	95.6% (95.30–95.82)	93.7% (93.28–94.08)	91.0% (90.57–91.38)	91.6% (91.23–91.97)	1–4
5	EQ-VAS	97.0% (96.86–97.22)	96.7% (96.55–96.95)	93.9% (93.58–94.24)	94.6% (94.27–94.85)	1–5
6	Osteoporosis	97.7% (97.68–97.94)	97.7% (97.72–97.98)	96.5% (96.80–97.22)	96.9% (97.01–97.39)	1–6
7	K-ADL	97.8% (97.81–98.04)	97.7% (97.79–98.04)	97.1% (97.37–97.72)	97.3% (97.47–97.79)	1–7
8	ABC	97.9% (97.90–98.10)	97.8% (97.89–98.10)	97.5% (97.88–98.10)	97.7% (97.88–98.10)	1–8
9	Fear of falling	97.9% (97.90–98.10)	97.8% (97.90–98.10)	97.5% (97.90–98.10)	97.7% (97.90–98.10)	1–9

ABC, activities-specific balance confidence; EQ-VAS, EuroQol visual analogue scale; K-ADL, Korean activities of daily living; PF, physical frailty; PF-M, physical frailty, and mobility; SARC-F, five-component Strength, Assistance with walking, Rising from a chair, Climbing stairs, and Falls; SGDS-K, Korean version of the short form geriatric depression scale; 95% CI, 95% confidence intervals

The performance of the model stabilized and demonstrated effectiveness when four variables were included, achieving all performance metrics above 95%. Adding more variables did not result in significant improvements in model performance. Therefore, the four variables, namely, the number of Fried PF phenotypes, PF-M score, SGDS-K score, and SARC-F score, were determined as the optimal variables. The logistic regression equation for the model with the optimal variables is as follows.


$$\text{ln}(\frac{\text{p}(\text{X})}{\text{1}-\text{p}(\text{X})})=\beta_{\text{0}}+\beta_{\text{1}}{\text{X}}_{\text{1}}+\beta_{\text{2}}{\text{X}}_{\text{2}}+\beta_{\text{3}}{\text{X}}_{\text{3}}+\beta_{\text{4}}{\text{X}}_{\text{4}}$$ (1)

where *P*(X) represents the probability of non-falls or falls ranging from 0 to 1, and X_1_, X_2_, X_3_, and X_4_ correspond to the number of Fried PF phenotypes, PF-M scores, SGDS-K scores, and SARC-F scores, respectively. β_0_ is the intercept (2.513), while β_1_–_4_ are the corresponding coefficients (β_1 _= −1.202, β_2 _= −0.004, β_3 _= −0.035, and β_4 _= −0.020, respectively).

## Discussion

In this study, we evaluated the influence of demographics, clinical conditions, and health status (i.e., physical and psychological health conditions) on fall risk in older adults with CF by developing, implementing, and validating a machine-learning framework. Overall, 443 older adults with CF (approximately 18.43%) were consistent with the reported global prevalence range of older adults with CF [34], confirming the robustness of the data set used in this study. Using data from the Korean Frailty and Aging Cohort Study (KFACS) and leveraging the machine learning approach, we developed a logistic regression-based model for predicting fall risk 2 years after the baseline assessment. This model demonstrated excellent predictive performance (AUC, sensitivity, specificity, and accuracy ≥96%) when including all nine significant variables (Table 3). Furthermore, our machine learning framework identified four optimal variables (i.e., Fried PF phenotypes, PF-M, SGDS-K, and SARC-F scores) that enabled accurate fall risk prediction, which demonstrated excellent predictive performance with an AUC ≥ 95%, sensitivity ≥93%, specificity ≥91%, and accuracy ≥91%. These findings confirm the efficacy of the machine learning approach combined with multidimensional health data (i.e., clinical condition and physical and psychological health) in accurately predicting fall risk.

The statistical analysis results for demographics and clinical characteristics indicated that osteoporosis was significantly different between the non-faller and faller groups. This result is consistent with previous studies showing that osteoporosis is associated with an increased risk of falling, particularly in older adults with reduced musculoskeletal impairments [7,8]. Although no statistically significant differences were observed in other clinical conditions, such as cardiovascular, respiratory, and cognitive disorders, this may be partly due to the small number of participants with these conditions, which could have limited the statistical power to detect true differences. In contrast, musculoskeletal deficits, particularly those related to osteoporosis, showed a significant association with fall risk, highlighting the need for targeted interventions in older adults with cognitive frailty. However, while osteoporosis was significantly associated with fall risk, the KFACS data did not include information on participants’ treatment status, medication adherence, or follow-up care related to osteoporosis. Therefore, the potential modifying effects of clinical management on fall risk could not be evaluated.

In addition, eight significant health-related variables were determined to be associated with falls in older adults with CF, including five physical health variables (number of Fried PF phenotypes and scores from K-ADL, PF-M, SARC-F, and ABC assessments) and three psychological health variables (EQ-VAS, SGDS-K, and fear of falling scores). The faller group had significantly greater levels of PF, with a higher number of Fried PF phenotypes and higher K-ADL and SARC-F scores than the non-faller group. Additionally, this group showed reduced mobility and balance confidence, as evidenced by the low PF-M and ABC scores. These findings are consistent with previous studies showing that balance and mobility deficits contribute to an increased fall risk in older adults [7,8]. Similarly, the faller group had high distress levels, as evidenced by high SGDS-K, fear of falling, and low EQ-VAS scores. Thus, psychological conditions may increase fall risk and contribute to a feedback loop in which fear and anxiety worsen mobility and exacerbate vulnerability to falls in older adults with CF [9,10]. Altogether, our results confirm the association between physical and psychological health conditions, suggesting a need for integrated fall prevention strategies to address both domains. For instance, combining balance training with psychological support could potentially reduce the compounded risks faced by older adults with CF [11,12].

The machine learning-based model developed in this study demonstrated good predictive performance. Specifically, the AUC exceeded 85% for all nine variables and four optimal variables (Table 3). These results confirm the robustness and reliability of the model for predicting fall risk in older adults with CF. Furthermore, the machine learning-based model with optimal variables (i.e., Fried PF phenotypes and PF-M, SGDS-K, and SARC-F scores) demonstrated excellent predictive performance, with an AUC, sensitivity, specificity, and accuracy exceeding 91%. These findings confirm the importance of both physical and psychological health conditions in predicting falls in older adults with CF. Fried PF phenotypes quantify impairments in five domains: unintentional weight loss, exhaustion, weakness, slow walking speed, and low physical activity [16]. An increase in the number of Fried PF phenotypes indicates greater overall PF, which has also served as a biomarker of vulnerability to falls in older adults [35,36]. The PF-M score, resulting from assessments of mobility and balance, reflects functional limitations in tasks critical for maintaining stability, such as walking, standing, and transitioning from a seated to a standing position. Mobility limitations hinder physical independence and increase the likelihood of falls during routine activities, particularly in older adults with CF, who often exhibit compromised neuromotor function [37,38]. The SGDS-K score evaluates depressive symptoms, which have been shown to negatively affect balance, movement coordination, and gait stability while exacerbating the fear of falling [9,10]. Its inclusion as an optimal variable emphasizes the important role of depression in fall risk prediction, a critical factor that remains underrecognized in older adults [39,40]. The SARC-F score assesses sarcopenia (age-related loss of muscle mass and strength), which captures the primary aspects of physical functionality [20]. Sarcopenia in older adults impairs their ability to perform essential daily tasks, such as climbing stairs or transitioning from a seated to a standing position, while limiting effective recovery from postural instability, thereby increasing the risk [8,20,41]. Taken together, the identified optimal variables highlight the multidimensional nature of fall risk prediction in older adults with CF. Therefore, the excellent predictive performance of the model with these optimal variables indicates its sufficiency in accurately predicting the fall risk without the need for additional variables. This finding offer practical advantages, as it simplifies the data collection process and facilitating the implementation of our study outcomes in clinical settings.

While this study provides valuable insights into the association between falls and CF and suggests a robust machine-learning-based model for accurately predicting fall risk, four limitations need to be acknowledged. First, we used data sets from a single cohort, which may limit the generalizability of the findings to other populations, including older adults with neurological disorders such as stroke or Parkinson’s disease. Second, the population in this study, comprising older Korean adults, may limit the applicability of our results and outcomes to populations with diverse demographic and cultural backgrounds owing to the homogeneity of the data sets. Third, although our machine learning-based model demonstrated excellent predictive performance, it was developed and validated using longitudinal data sets spanning 2 years. Fourth, although the study included participants from diverse geographic regions across South Korea, including urban, suburban, and rural areas, the model did not explicitly account for environmental or contextual factors such as walkability, infrastructure, or climate. These unmeasured variables may influence fall risk and act as potential confounders. Therefore, caution is warranted when applying the model to populations with markedly different environmental exposures. Therefore, our future research will prioritize validating the model developed in this study across multiple cohorts, including those with not only diverse demographic characteristics but also varying residential and environmental contexts, such as differences in urbanization level, neighborhood infrastructure, and access to community resources, to better assess the model’s generalizability across diverse populations and settings. In addition, our future work will integrate explainable artificial intelligence techniques to enhance model interpretability and expand the modeling framework to include longitudinal features such as fall history and evolving risk factors. This approach may enable health professionals to understand the contributions of individual predictors over time and support the broader clinical adoption of personalized fall risk prediction tools.

## Conclusion

This study presents a robust and innovative approach for advancing fall risk prediction in older adults with CF by incorporating machine-learning methodologies with multidimensional health data. Using 2-year longitudinal data, we developed and validated a machine-learning-based model that demonstrated excellent predictive performance with high AUC, sensitivity, specificity, and accuracy. Our machine-learning framework identified four optimal variables that offer a simplified yet highly effective method for accurate fall risk prediction. Thus, the model has the potential to serve as a reliable tool for clinical decision-making, advancing beyond traditional statistical methods in identifying older adults within the CF population who are at high risk of falling.

Our findings have practical implications for fall prevention and management in older adults with CF. The optimal variables identified in this study can allow healthcare professionals to focus on key assessments and improve the efficiency of clinical evaluations without compromising their accuracy. Additionally, the model developed and validated in this study can be applied in diverse healthcare settings, such as community clinics and geriatric healthcare centers, to facilitate early interventions tailored to the unique needs of older adults with CF. Moreover, the findings of this study suggest the potential for the future development of fall risk monitoring systems that can integrate real-time data for continuous risk assessment in older adults with CF.
